# Distinction of *ALK* fusion gene‐ and *EGFR* mutation‐positive lung cancer with tumor markers

**DOI:** 10.1111/1759-7714.15268

**Published:** 2024-02-24

**Authors:** Takahiro Akita, Ryo Ariyasu, Sho Kakuto, Keiki Miyadera, Ayu Kiritani, Ryosuke Tsugitomi, Yoshiaki Amino, Ken Uchibori, Satoru Kitazono, Noriko Yanagitani, Sadatomo Tasaka, Makoto Nishio

**Affiliations:** ^1^ Department of Thoracic Medical Oncology The Cancer Institute Hospital, Japanese Foundation for Cancer Research Tokyo Japan; ^2^ Department of Respiratory Medicine Hirosaki University Graduate School of Medicine Hirosaki Japan

**Keywords:** ALK‐positive lung cancer, CEA, CYFRA21‐1, EGFR‐positive lung cancer, tumor marker

## Abstract

**Background:**

It is difficult to predict gene mutations individually based on clinical background alone. Tumor markers may help to predict each gene mutation. Identifying tumor markers that can predict gene mutation will facilitate timely genetic testing and intervention.

**Methods:**

We selected 134 cases of advanced or recurrent ALK‐positive and 172 cases of advanced or recurrent EGFR‐positive lung cancer from our clinical database. The cutoff values for the tumor markers were defined as 5.0 ng/mL or higher for carcinoembryonic antigen (CEA) and 3.5 ng/mL or higher for soluble fragment of cytokeratin 19 (CYFRA21‐1) in accordance with the institutional standards. A positive CYFRA21‐1:CEA ratio was defined as 0.7 or higher.

**Results:**

The CEA‐positivity rate was 49% for ALK‐positive lung cancers and 73% for EGFR‐positive lung cancers, which was significantly different (*p* < 0.001). The CYFRA21‐1 positivity rate was significantly higher in ALK‐positive lung cancer (36%) compared with EGFR‐positive lung cancer (23%) (*p* = 0.034). The median CYFRA21‐1:CEA ratio was 0.395 for the ALK group, which was significantly higher compared with 0.098 for the EGFR group (*p* < 0.001). These trends were similar when excluding histology other than adenocarcinoma. The median time‐to‐treatment failure (TTF) for initial tyrosine kinase inhibitor (TKI) therapy was 308 days for the high CYFRA21‐1:CEA ratio group and 617 days for the low CYFRA21‐1:CEA ratio group for ALK‐positive lung cancer (*p* = 0.100).

**Conclusions:**

A higher proportion of patients with ALK‐positive lung cancer were CYFRA21‐1 positive and had higher CYFRA21‐1:CEA ratios compared with EGFR‐positive lung cancer patients.

## Graphical abstract

The median CYFRA21‐1:CEA ratio was 0.395 for the ALK‐positive lung cancer group, which was significantly higher compared with 0.098 for the EGFR‐positive lung cancer group. These trends were similar when excluding histology other than adenocarcinoma.

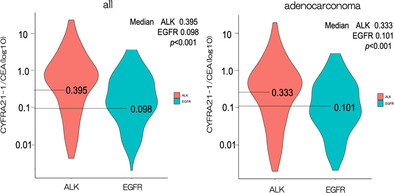

## INTRODUCTION

Lung cancer remains the leading cause of cancer‐related deaths and has poor prognosis; however, treatment outcomes of non‐small cell lung cancer (NSCLC) have improved dramatically with the approval of molecular targeted drugs and the discovery of driver gene mutations. Targeting driver gene mutations with specific drugs has been adopted as standard therapy,^1^ and the prognosis of NSCLC patients has markedly improved over the last decade,^2^ highlighting the importance of driver gene mutations in patients with NSCLC. The most common driver gene mutations identified in NSCLC are *EGFR* mutations and the *ALK* fusion gene. *EGFR* mutations (EGFR positive) in NSCLC are frequent among Asian populations and account for 40%–50% of NSCLC cases and approximately 20% among Caucasian patients.^3^ NSCLC with an *ALK* fusion gene (ALK‐positive) is the second most common type and accounts for 3%–5% of all cases.^4^, ^5^

The prognosis of patients with EGFR‐ and ALK‐positive NSCLC has significantly improved following treatment with EGFR tyrosine kinase inhibitors (EGFR‐TKIs) and ALK–TKIs, respectively. In particular, ALK‐positive NSCLC has a 5‐year survival rate of 60% with ALK–TKI therapy; therefore, it is important detect the *ALK* fusion gene in NSCLC patients.^6^ Driver gene mutations are detected using comprehensive gene panel tests (Foundation one, Oncomine CDX, AmoyDX and so on) or singleplex assays, which include real‐time polymerase chain reaction (RT‐PCR) for EGFR and fluorescent in situ hybridization (FISH), immunohistochemistry (IHC), and RT‐PCR for ALK. It is desirable to analyze all NSCLC patients by these methods; however, in actual clinical practice, it is not possible to analyze gene mutations in every case because of insufficient tissue samples and cost. Moreover, there is a sensitivity issue with gene testing. A study showed that the sensitivity and specificity of ALK‐IHC referred to by NGS were 75.0% and 98.7%, respectively.^7^

Clinical background may help to predict driver gene mutations. For example, patients with driver gene‐positive NSCLC have been reported to be younger individuals without a smoking history.^8^, ^9^ However, because these clinical backgrounds are common to both ALK‐ and EGFR‐positive NSCLC, it is difficult to predict gene mutations by clinical background alone. Therefore, additional clinical markers are needed to distinguish ALK‐ and EGFR‐positive NSCLC.

Tumor markers are used in clinical practice as an adjunctive diagnosis to predict efficacy of treatment, recurrence, and metastasis. The positivity rate of tumor markers increases with disease stage.^10^, ^11^ Many studies have found serum tumor marker levels to have prognostic value.^12^ Tumor markers are easy to use because samples can be collected by blood sampling. The positive rate of tumor markers differs depending on the histologic type and degree of differentiation. In general, CEA is used as a marker for adenocarcinoma and CYFRA21‐1 tends to be positive in squamous cell carcinoma.^13^, ^14^ ALK‐ and EGFR‐positive NSCLCs have distinct histological profiles,^15^, ^16^, ^17^ suggesting that their tumor marker profiles may also be different; however, no study to date has compared the tumor marker profile of NSCLCs with different driver gene mutations.

In the present study we aimed to identify tumor markers specific for ALK‐positive NSCLC by comparing tumor marker levels between patients with ALK‐positive and EGFR‐positive NSCLC. If we found features for ALK‐positive NSCLC, we believed the tumor marker may predict ALK‐positive NSCLC with reference to patient background.

## METHODS

### Study design and patient population

In the present study, we compared tumor marker expression between patients with ALK‐positive and EGFR‐positive lung cancer. In addition, we compared the clinical features of patients categorized based on tumor marker expression. The database of the Department of Respiratory Medicine, Cancer Institute Hospital of the Japanese Foundation for Cancer Research, was used to select the cases of advanced or recurrent ALK‐positive lung cancer between January 2009 and August 2022. For EGFR‐positive lung cancer, advanced or recurrent cases were selected as the control group in order of the patient registration number between July 2018 and August 2022. We included all eligible patients with ALK‐positive lung cancer in the database as well as a comparable number of eligible patients with EGFR‐positive lung cancer. Some tumor markers tend to be elevated in specific histological types, for example, CYFRA21‐1 tends to be elevated in patients with squamous cell carcinoma,^14^ whereas CEA tends to be elevated in those with adenocarcinoma.^13^ To exclude the histological effects and to conclude only the driver genes effect, we also performed analyses specifically in patients with adenocarcinomas. Patient background, age, sex, histological type, smoking history, stage, metastatic disease, and presence of genetic mutations were evaluated. *EGFR* mutation positivity was confirmed by either Cobas EGFR version 2 or Oncomine Dx. *ALK* fusion gene positivity was confirmed by IHC, FISH, RT‐PCR, or Oncomine Dx. The values for the blood tumor markers, CEA, and CYFRA21‐1 were extracted from medical records. Tumor markers in the blood were measured before treatment at our hospital. CEA and CYFRA21‐1 levels were measured with clinical tests approved by the Pharmaceuticals and Medical Devices Agency. CEA levels were measured using the Alinity I CEA (Abbott Laboratories) chemiluminescence immunoassay. CYFRA21‐1 levels were measured using the Lumipulse Presto CYFRA (FUJIREBIO) chemiluminescent enzyme immunoassay. The cutoff values for CEA and CYFRA21‐1 were 5.0 ng/mL and 3.5 ng/mL, respectively. These values were determined based on the manufacturer's instructions that describe the normal range of tumor markers in healthy individuals and the community standard in Japan, which was established using controls from patients with benign diseases. These cutoff values were used to derive the CYFRA21‐1:CEA ratio.

The present study was approved by the Institutional Review Board of Cancer Institute Hospital of Japanese Foundation for Cancer Research (approval no. 2022‐GB‐165). Informed consent was waived because of the retrospective nature of the study, and an opt‐out option was included.

### Statistical analysis

A Pearson's chi‐square test was used to compare proportions between the two groups and a Mann–Whitney U test was used for median comparisons. Time‐to‐treatment failure (TTF) for ALK–TKI and EGFR–TKI was evaluated using the Kaplan–Meier method. TTF was defined as the period from the start date of ALK–TKI and EGFR–TKI therapy to the end of treatment. A *p*‐value less than 0.05 was considered statistically significant. Statistical analyses were performed using SPSS Statistics 24 software.

## RESULTS

### Patient characteristics

**FIGURE 1:**
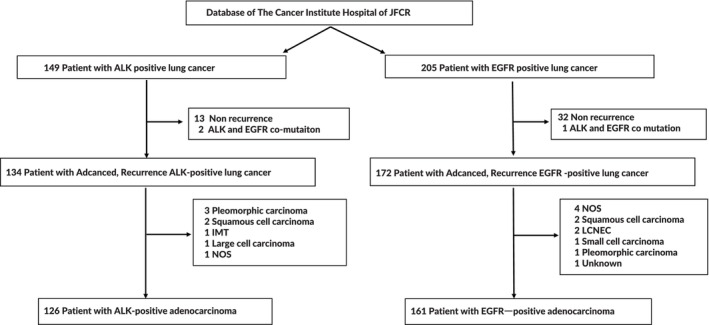
Patient selection flow chart. ALK‐positive and EGFR‐positive lung cancers were selected from the database of The Cancer Institute Hospital of JFCR. A total of 134 patients with ALK‐positive lung cancer and 172 patients with EGFR‐positive lung cancer were selected after excluding postoperative recurrence‐free and comutation of ALK‐positive and EGFR‐positive cancers. Furthermore, narrowed down to adenocarcinoma, 126 patients with ALK‐positive lung cancer and 161 patients with EGFR‐positive lung cancer were selected. ALK, anaplastic lymphoma kinase; EGFR, epidermal growth factor receptor.

The database contained 149 ALK‐positive lung cancer patients and analyses were performed on 134 patients with advanced or recurrent ALK‐positive lung cancer, which excluded recurrence‐free cases and comutations with EGFR. Of 205 EGFR‐positive lung cancer patients, 172 cases of advanced or recurrent EGFR‐positive lung cancer were analyzed, excluding recurrence‐free cases and comutations with ALK. In addition, 126 ALK‐positive and 161 EGFR‐positive lung adenocarcinoma patients, excluding nonadenocarcinoma patients, were also analyzed (Figure 1).

The ALK and EGFR groups included 134 and 172 patients, respectively. Patient characteristics are summarized in Table 1. In the overall cohort, the median CEA level was 4.7 ng/mL in the ALK group and 18.3 ng/mL in the EGFR group, and the difference between the two groups was significant (*p* = 0.001). The median CYFRA21‐1 level was 2.0 ng/mL in the ALK group, which was significantly higher than that in the EGFR group (1.7 ng/mL; *p* = 0.004). In analyses of only those patients with adenocarcinoma, the median CEA level was 5.2 ng/mL in the ALK group and 17.3 ng/mL in the EGFR group, with a statistically significant difference (*p* = 0.007). The median CYFRA21‐1 level was 2.0 ng/mL in the ALK group, which was significantly higher than that in the EGFR group (1.6 ng/mL; *p* = 0.023) (Table 1).

**TABLE 1:** Patient characteristics.

Characteristics	All			Adenocarcinoma		
	ALK (*n* = 134)	EGFR (*n* = 172)	*p*‐value	ALK (*n* = 126)	EGFR (*n* = 161)	*p*‐value
	*N* (%)	*N* (%)		*N* (%)	*N* (%)	
Age						
<65	105 (78)	72 (42)	<0.001	98 (78)	66 (41)	<0.001
≥65	29 (22)	100 (58)		28 (22)	95 (59)	
Sex						
Male	69 (51)	69 (40)	0.047	62 (49)	62 (39)	0.079
Female	65 (49)	103 (60)		64 (51)	99 (61)	
Smoking status						
Current or former	54 (40)	75 (44)	0.561	49 (39)	67 (42)	0.686
Never	80 (60)	97 (56)		77 (61)	94 (58)	
Stage						
III/IV + recurrence	4/130 (3/97)	3/169 (2/98)	0.471	4/122 (3/97)	3/158 (2/98)	0.475
CEA median(ng/mL)	4.7	18.3	0.001	5.2	17.3	0.007
	*n* = 114	*n* = 165		*n* = 107	*n* = 155	
CYFRA21‐1 median (ng/mL)	2.0	1.7	0.004	2.0	1.6	0.023

Abbreviations: ALK, anaplastic lymphoma kinase rearrangement; CEA, carcinoembryonic antigen; CYFRA21‐1, soluble fragment of cytokeratin 19; EGFR, epidermal growth factor receptor; Stage, clinical stage based on the TNM classification; Recurrence, postoperative recurrence cases.

### Trends in CEA and CYFRA21‐1 between ALK‐positive and EGFR‐positive patients

**FIGURE 2:**
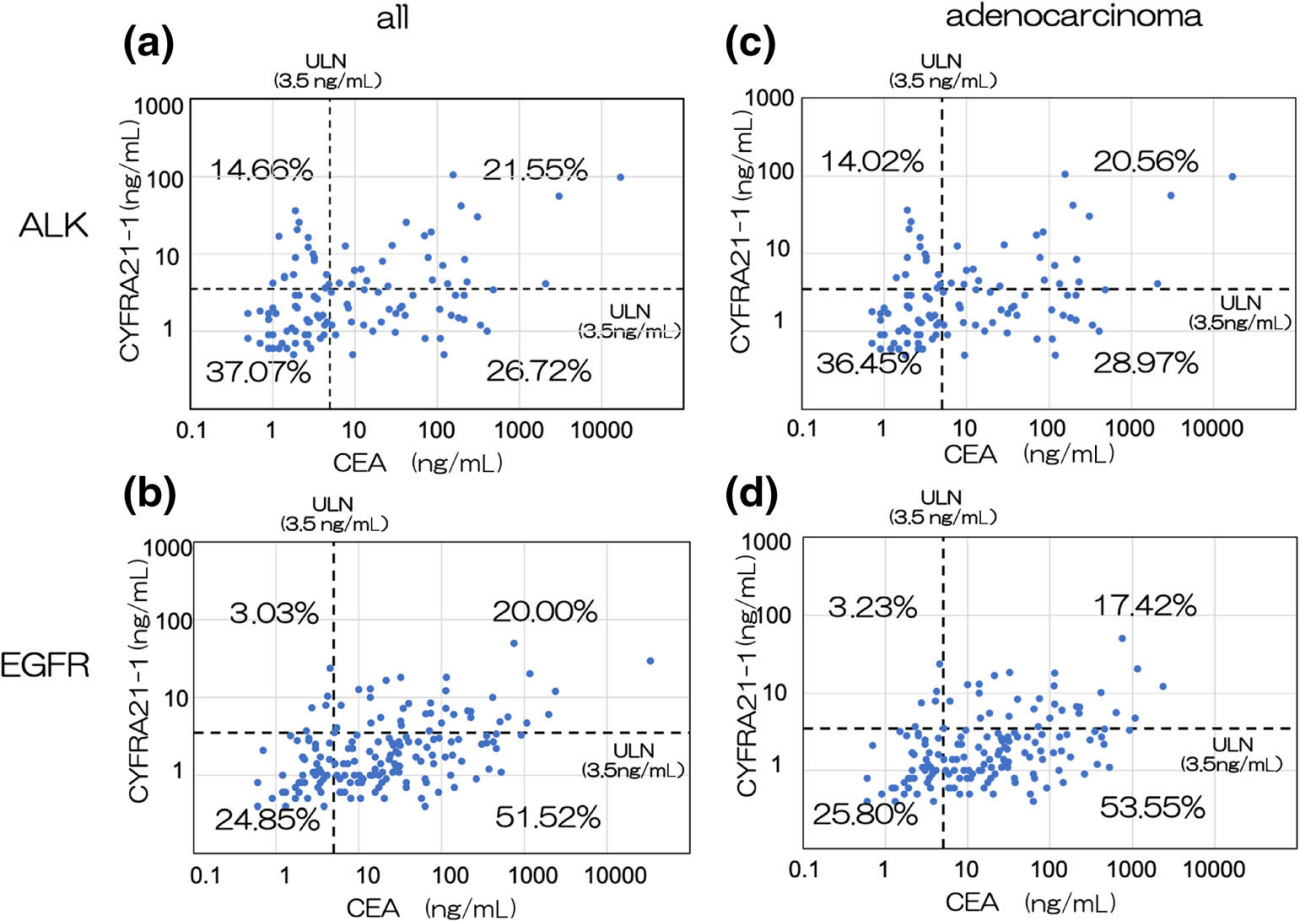
Plots of the trends of carcinoembryonic antigen (CEA) and soluble fragment of cytokeratin 19 (CYFRA21‐1) in the anaplastic lymphoma kinase (ALK) and epidermal growth factor receptor (EGFR) groups. (a) Trend of CEA and CYFRA21‐1 in the ALK group and (b) trend of CEA and CYFRA21‐1 in the EGFR group. (c) Trend of CEA and CYFRA21‐1 in the ALK group and (d) trend of CEA and CYFRA21‐1 in the EGFR group with the histological type restricted to adenocarcinoma.

In the ALK group, 49% of the cases were CEA‐positive cases, which was significantly lower compared with 73% for the EGFR group (*p* < 0.001). There were 36% and 23% positive CYFRA21‐1 cases in the ALK and EGFR groups, respectively, and the difference was significant (*p* = 0.034). For adenocarcinoma cases only, CEA positivity was also significantly lower in the ALK group (49%) compared with 71% in the EGFR group (*p* < 0.001). CYFRA21‐1‐positive cases in the ALK and EGFR group were 35% and 21%, respectively, and the difference was significant (*p* = 0.012) (Figure S1). The distribution of CYFRA21‐1 and CEA in the ALK and EGFR groups is shown in Figure 2. In the ALK group, 14.66% of the patients were CYFRA21‐1‐positive and CEA‐negative, which was higher than that in the EGFR group (3.03%). The same trend was observed in adenocarcinoma patients.

### CYFRA21‐1:CEA ratio

**FIGURE 3:**
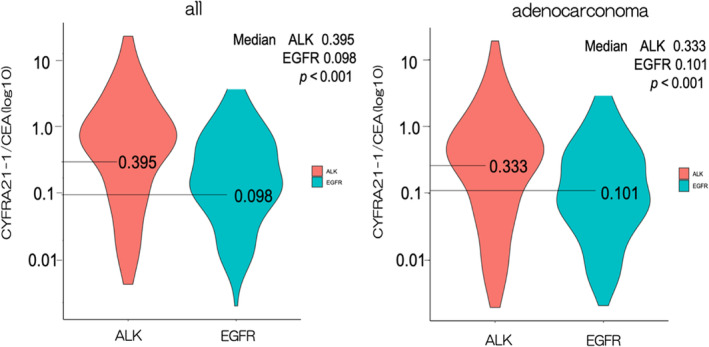
Trends of soluble fragment of cytokeratin 19:carcinoembryonic antigen (CYFRA21‐1:CEA) ratio in the anaplastic lymphoma kinase (ALK) and epidermal growth factor receptor (EGFR) groups. (a) The distribution of the CYFRA21‐1:CEA ratio in the ALK and EGFR groups is shown in the violin plot. (b) The distribution of the CYFRA21‐1:CEA ratio in the ALK group and EGFR groups representing adenocarcinoma cases is shown in the violin plot.

Tumor marker levels tend to be higher in larger tumors. Therefore, to exclude the potential effect of differences in tumor volume on changes in tumor marker levels, we also compared the CYFRA21‐1:CEA ratio between the groups. The distribution of the CYFRA21‐1:CEA ratio in the ALK and EGFR groups is shown in Figure 3. The median of the CYFRA21‐1:CEA ratio was 0.395 in the ALK group and 0.098 in the EGFR group, which was significantly different (*p* < 0.001). The same trend was observed for the adenocarcinoma cases (0.333 in the ALK group and 0.101 in the EGFR group; *p* < 0.001). The cutoff value for the CYFRA21‐1:CEA ratio was defined as 0.7 (3.5 /5.0) using the cutoff values for each tumor marker. The percentage of patients with a high CYFRA21‐1:CEA ratio was 29% in the ALK group and 14% in the EGFR group, which was significantly different (*p* = 0.002). The percentage of patients with a high CYFRA21‐1:CEA ratio was also significantly higher in the ALK group (28%) compared with the EGFR group (14%) in cases restricted to adenocarcinoma (*p* = 0.004) (Figure 3).

### Clinical characteristics of patients with high and low CYFRA21‐1:CEA ratios

We determined whether there were differences between patients with high and low CYFRA21‐1:CEA ratios based on clinical characteristics and whether there was a difference in the efficacy of ALK‐TKI and EGFR‐TKI therapy. In the present study, we defined a CYFRA21‐1:CEA ratio of ≥0.7 as high and a CYFRA21‐1:CEA ratio of <7 as low. For patients with high and low CYFRA21‐1:CEA ratios, there were no differences in age, gender, or smoking history in either the ALK or EGFR group (Table 2). The same trend was observed in strictly adenocarcinoma cases.

**TABLE 2:** Comparison of clinical characteristics of patients with ALK‐ and EGFR‐positive lung cancer categorized according to the CYFRA21‐1:CEA ratio.

Characteristics	ALK			EGFR		
	CYFRA21‐1/CEA			CYFRA21‐1/CEA		
	≥0.7	<0.7		≥0.7	<0.7	
	*N* = 35	*N* = 79	*p*‐value	*N* = 23	*N* = 142	*p*‐value
Age						
<65	29 (83)	59 (75)	0.337	13 (57)	53 (37)	0.338
≥65	6 (17)	20 (25)		10 (43)	89 (63)	
Sex						
Male	21 (60)	41 (52)	0.423	9 (39)	57 (40)	0.927
Female	14 (40)	38 (48)		14 (61)	85 (60)	
Smoking status						
Current or former	15 (43)	31 (39)	0.717	11 (48)	61 (43)	0.662
Never	20 (57)	48 (61)		12 (52)	81 (57)	
Stage						
III/IV + recurrence	2/33 (6/94)	1/78 (1/99)	0.171	1/22 (4/96)	2/140 (1/98)	0.328
Histology						
Ad/others	31/4 (89/11)	76/3 (96/4)	0.117	21/2 (91/9)	134/8 (94/6)	0.568

Abbreviations: Ad, adenocarcinoma; ALK, anaplastic lymphoma kinase; CEA, carcinoembryonic antigen; CYFRA21‐1, soluble fragment of. cytokeratin 19; EGFR, epidermal growth factor receptor.

Initial TKI treatment efficacy in patients with a high and low CYFRA21‐1:CEA ratio was evaluated by TTF. Overall, 101 patients were treated with ALK–TKI and 143 patients were treated with EGFR–TKI. The TKIs used for the ALK–TKI were alectinib (57 patients), crizotinib (35 patients), certinib (6 patients), lorlatinib (3 patients). osimertinib (129 patients), gefitinib (6 patients), afatinib (6 patients), erlotinib (2 patients) were used as EGFR–TKIs. For the ALK group, the median TTF was 308 days in cases exhibiting a high CYFRA21‐1:CEA ratio and 617 days in cases with a low CYFRA21‐1:CEA ratio (*p* = 0.100). In the EGFR group, the median TTF was 259 days in cases with a high CYFRA21‐1:CEA ratio, which was significantly shorter compared with 598 days in cases with a low CYFRA21‐1:CEA ratio (*p* = 0.002).

**FIGURE 4:**
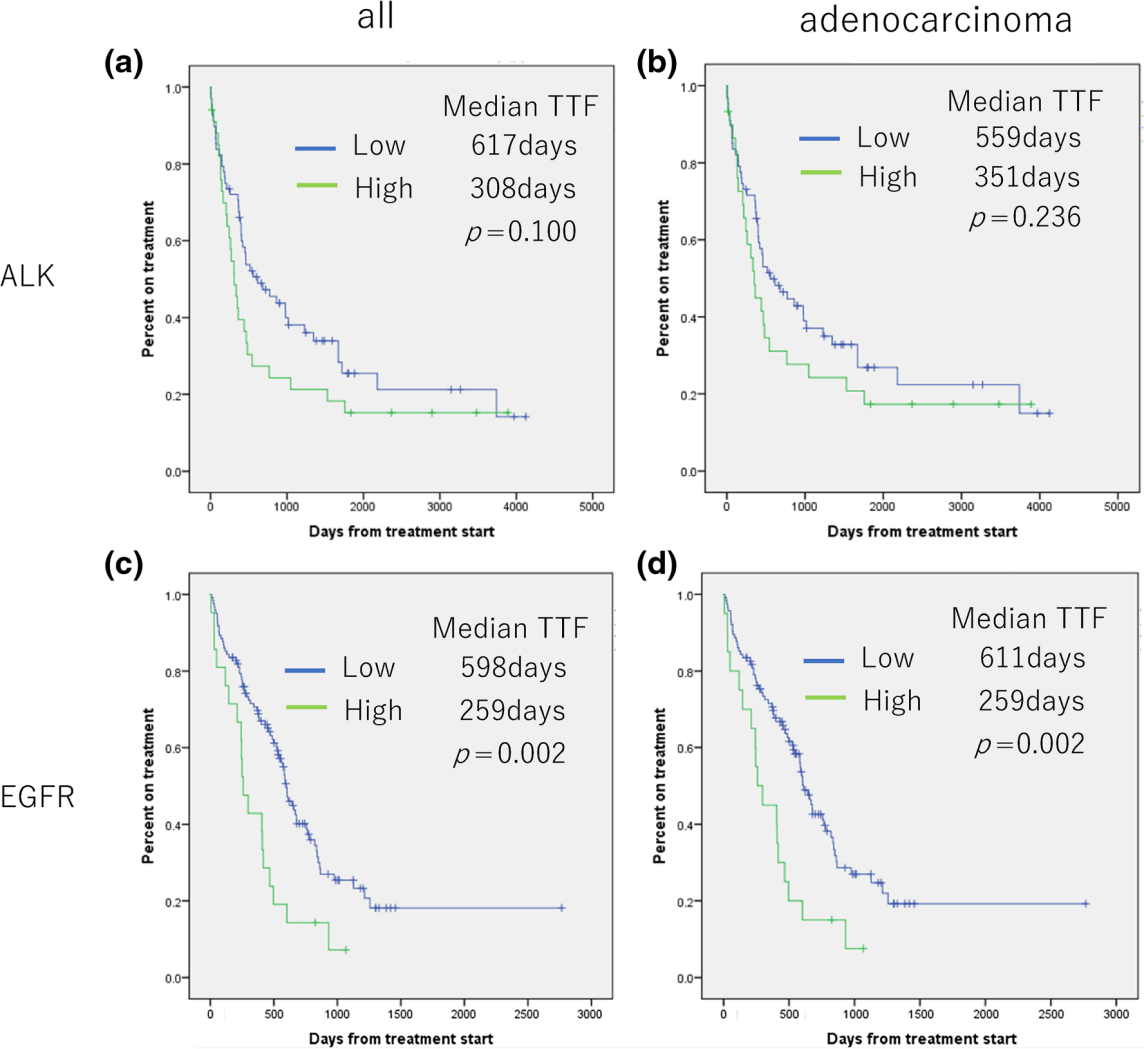
The time‐to‐treatment failure (TTF) of the first tyrosine kinase inhibitor (TKI) was evaluated using the Kaplan–Meier method. Patients were divided into groups based on a soluble fragment of cytokeratin 19:carcinoembryonic antigen (CYFRA21‐1:CEA) ratio greater than or equal to 0.7. (a) Kaplan–Meier curves of TTF in the anaplastic lymphoma kinase (ALK) group, and (b) Kaplan–Meier curves of TTF in the epidermal growth factor receptor (EGFR) group without distinguishing the histological type. (c) and (d) Kaplan–Meier curves for TTF in the ALK and EGFR adenocarcinoma groups, respectively.

The same analysis was done for the adenocarcinoma cases, in which 96 patients received ALK–TKIs and 135 patients received EGFR–TKIs. The TKIs used in the ALK–TKI group were alectinib (53 patients), crizotinib (34 patients), certinib (6 patients), and lorlatinib (3 patients). For the EGFR–TKI group, the TKIs included osimertinib (122 patients), gefitinib (6 patients), afatinib (5 patients), and erlotinib (2 patients). In the ALK group, the median TTF with a high CYFRA21‐1:CEA ratio was 351 days, and 559 days (*p* = 0.236) for a low CYFRA21‐1:CEA ratio. In the EGFR group, the median TTF with a high CYFRA21‐1:CEA ratio was 259 days, which was significantly shorter compared with 611 days for cases with a low CYFRA21‐1:CEA ratio (*p* = 0.002) (Figure 4).

### Impact of tumor marker values on PS and distant metastasis in ALK‐positive and EGFR‐positive lung cancer

We investigated the impact of tumor marker values on performance status (PS) and distant metastasis in patients with ALK‐positive and EGFR‐positive lung cancer. Our analysis showed that patients with elevated tumor markers tended to have poorer PS and more visceral metastases. In ALK‐positive patients, we found significant associations between poor PS and bone metastasis with CYFRA 21‐1 levels. Similarly, bone metastasis was associated with CEA levels while poor PS, brain, and bone metastases were associated with CYFRA21‐1 levels in EGFR‐positive patients (Table S2). Moreover, we observed that higher levels of CYFRA21‐1 were associated with a shorter TTF, consistent with the trend observed with the CYFRA21‐1:CEA ratio (Figure S1).

## DISCUSSION

In the present study, more ALK‐positive lung cancers were CYFRA21‐1‐positive with higher CYFRA21‐1:CEA ratios compared with EGFR‐positive lung cancers. CYFRA21‐1 is generally a marker for squamous cell carcinoma. It is not considered useful in ALK‐ and EGFR‐positive lung cancers because most are adenocarcinomas. However, in the present study, 36% of ALK‐positive lung cancer patients were unexpectedly CYFRA21‐1‐positive. Furthermore, the ratio of CYFRA21‐1 and CEA was significantly different between ALK‐ and EGFR‐positive NSCLC. The present analysis, which was limited to adenocarcinomas, still shows that ALK‐positive lung cancer is more likely to be positive for CYFRA21‐1, suggesting that it is not due to the presence of squamous cell carcinoma.

ALK‐ and EGFR‐positive lung cancers are predominantly adenocarcinomas, but there are differences in pathological subtypes. Most cases of EGFR‐positive lung cancer are of the lepidic element‐dominant histological subtype.^15^ On the other hand, ALK‐positive lung cancer was reported to consist predominantly of acinar and solid types.^16^, ^17^ These histological differences may affect the tumor marker profile. Terada et al. reported that cytokeratin 5 (CK5), a marker of squamous cell carcinoma, was detected in lung adenocarcinomas.^18^ The CK5‐positivity rate was significantly higher in ALK‐positive lung adenocarcinomas compared with the wild‐type (WT) and was significantly lower in EGFR‐positive lung adenocarcinomas compared with the WT. Because CYFRA21‐1 measures fragments of cytokeratin, the different cytokeratin positivity rates may explain the difference in CYFRA21‐1 values in ALK‐ and EGFR‐positive lung cancer. Further research is warranted to explore and elucidate the underlying mechanisms responsible for the difference in CYFRA21‐1 values between ALK‐ and EGFR‐positive lung cancers. Smoking history influences the positivity rate of CEA and SCC; however, in the present study, there was no relationship between smoking history and CYFRA21‐1 positivity in ALK‐ or EGFR‐positive NSCLC.

Wang et al. previously evaluated tumor markers in ALK‐positive lung cancers.^19^ They compared CYFRA21‐1 levels in 43 ALK‐positive patients with 641 WT patients and found no difference in CYFRA21‐1 positivity; however, the cutoff value for CYFRA21‐1 was 2.5 ng/mL, which was lower compared with that of the present study. We used the CYFRA21‐1:CEA ratio to avoid the effect of cutoff values and found that the ratio was higher in ALK‐positive compared with EGFR‐positive lung cancer. Moreover, they compared ALK‐positive lung cancer with WT cancers, which included various types of NSCLC, even with another mutation‐positive lung cancer, which could diminish the characteristics of each driver mutation. We compared EGFR‐positive and ALK‐positive tumors in a relatively homogeneous group, which may have clarified the tumor marker difference. Moreover, we also compared the data with adenocarcinomas, a more homogenous group, which exhibited the same trend.

The *ALK* fusion gene is rare, but it is important to use it for stratifying NSCLC patients for ALK–TKI treatment. However, it is difficult to obtain sufficient tissue samples to perform all gene mutation tests in many cases. If ALK‐positive lung cancer can be predicted by tumor markers, such as CYFRA21‐1 positivity and the CYFRA21‐1:CEA ratio, we could prioritize the singleplex test for the *ALK* fusion gene at the initial stages of treatment.

We also examined the effect of ALK–TKI and EGFR–TKI based on the CYFRA21‐1:CEA ratio. In the ALK and EGFR groups, the median TTF was shorter when the CYFRA21‐1:CEA ratio was high. In the ALK group, the median TTF was significantly shorter in the group of CYFRA21‐1‐positive cases (Figure S2). These findings suggest that ALK–TKI may be less effective in CYFRA21‐1‐positive patients. Previous reports showed a similar trend, in which TKIs were less effective in squamous cell carcinoma, and CK5‐positive lung adenocarcinomas had a worse prognosis.^18^, ^20^ It is not clear why TKI efficacy is worse in CYFRA21‐1‐positive ALK lung cancer, but this will need to be examined in future studies, because many ALK‐positive lung cancers are positive for CYFRA21‐1, as shown in our study. Furthermore, our study found that patients with high tumor markers were more likely to have poor PS and visceral metastases. Therefore, it is imperative to identify the driver mutation and provide early drug interventions for these patients. A singleplex test with a short TAT may be beneficial in identifying *EGFR* or *ALK* mutations in patients with elevated specific tumor markers.

There were some limitations in this study. It was a single‐center study and there may have been differences depending on the tumor marker settings and measurement methods at each institution. Because the number of cases was small, it will be necessary to collect and analyze a larger number of cases. Selection bias is inevitable because of the retrospective nature of this study. Most advanced lung cancer patients were diagnosed based on a small biopsy sample; therefore, it is unclear whether the squamous cell carcinoma component was excluded in all of the adenocarcinoma cases. In this study, significant differences in age and sex were also observed between patients with ALK‐positive and EGFR‐positive lung cancers, although the effects of age and sex on tumor markers are unclear. As tumor markers are elevated in patients with cancer, the effect of the different driver mutations of cancer is likely to be more significant than the effect of age and gender. However, this study cannot completely exclude the influence of age and sex. Finally, the retrospective study design precluded the examination of potential underlying mechanisms and external validation analyses.

In conclusion, ALK‐positive lung cancer patients exhibit higher CYFRA21‐1 positivity and have higher CYFRA21‐1:CEA ratios compared with EGFR‐positive lung cancer patients. Tumor markers may predict individual driver mutations. If tumor markers can accurately predict ALK‐positive lung cancer, this would allow for the timely submission of appropriate genetic tests and early interventions. Additionally, it would be beneficial to conduct large‐scale studies to investigate other driver mutations.

## AUTHOR CONTRIBUTIONS

Takahiro Akita: Conceptualization, data curation, formal analysis, methodology, resources and writing–original draft. Ryo Ariyasu, Makoto Nishio: Conceptualization, methodology, resources, supervision, and writing–review and editing. Sho Kakuto, Keiki Miyadera, Ayu Kiritani, Ryosuke Tsugitomi, Yoshiaki Amino, Ken Uchibori, Satoru Kitazono, Noriko Yanagitani and Sadatomo Tasaka: Resources, writing–review and editing.

## CONFLICT OF INTEREST STATEMENT

R. Ariyasu reports honoraria from Chugai Pharmaceuticals, Bristol Myers Squib, AstraZeneca, outside the submitted work, K. Uchibori reports honoraria from AstraZeneca, Amgen, Chugai, Takeda Pharmaceutical, Ono Pharmaceutical, Novartis, Eli‐Lilly, Thermofisher, Bristol Myers Squib, Merck, Daiichi‐Sankyo outside the submitted work. S. Kitazono reports honoraria from AstraZeneca, Ono Pharmaceutical Chugai Pharmaceuticals and Pfizer outside the submitted work. N. Yanagitani reports honoraria from Chugai Pharmaceuticals, Bristol Myers Squib, Ono Pharmaceuticals, AstraZeneca, Eli Lilly, Takeda Pharmaceutical Company, Pfizer; payment for expert testimony from Chugai Pharmaceuticals outside the submitted work. S. Tasaka reports honoraria from Ono Pharmaceuticals, Chugai Pharmaceutical, Taiho Pharmaceutical, Sanofi, Daiichi Sankyo, Lilly, AstraZeneca, AbbVie, Takeda, Pfizer, Boehringer Ingelheim, Kyowa Kirin, Kyorin, GlaxoSmithKline outside submitted work. M. Nishio reports honoraria from Ono Pharmaceuticals, Bristol Myers Squibb, Daiichi Sankyo, Pfizer, Chugai Pharmaceuticals, Eli Lilly, Taiho Pharmaceutical, AstraZeneca, MSD, AbbVie, Boehringer‐Ingelheim, Novartis, Nippon Kayaku, Merck Biopharma, Takeda Pharmaceutical, Janssen outside the submitted work. The remaining authors have no conflicts of interest to declare.

## Supporting information


**DATA S1:** Supplementary Information.
