# Supplementary material for: Distinction of ALK fusion gene‐ and EGFR mutation‐positive lung cancer with tumor markers
Source: Thorac Cancer. 2024 Feb 24;15(10):788–96. doi: 10.1111/1759-7714.15268 (PMC10995710; doi:10.1111/1759-7714.15268)

**Supplementary material**

Supplementary Table 1

(A) Positive rates of CEA and CYFRA21-1 in ALK-positive and EGFR-positive lung cancer.

(B) Positive rates of CEA and CYFRA21-1 in ALK-positive and EGFR-positive lung adenocarcinomas.


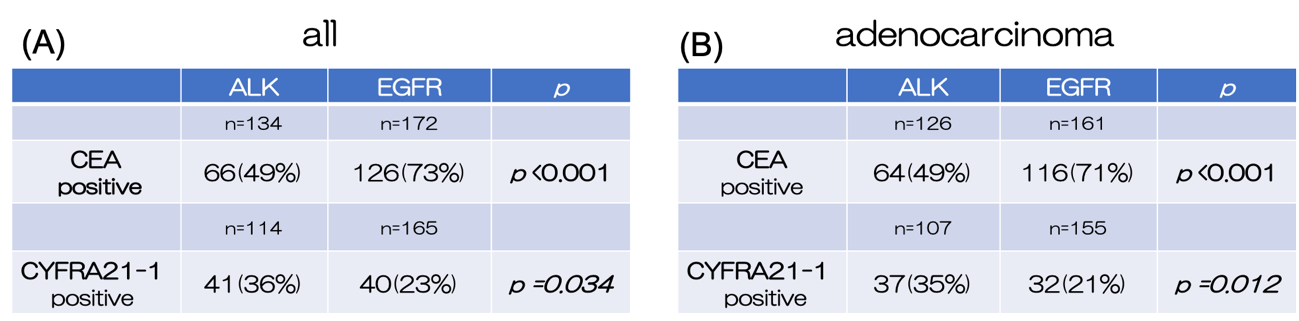


Supplementary Table

2a) Impact of tumor marker values on PS and distant metastasis in patients with ALK-positive lung cancer

2b) Impact of tumor marker values on PS and distant metastasis in patients with EGFR-positive lung cancer

Supplementary Table 2a)


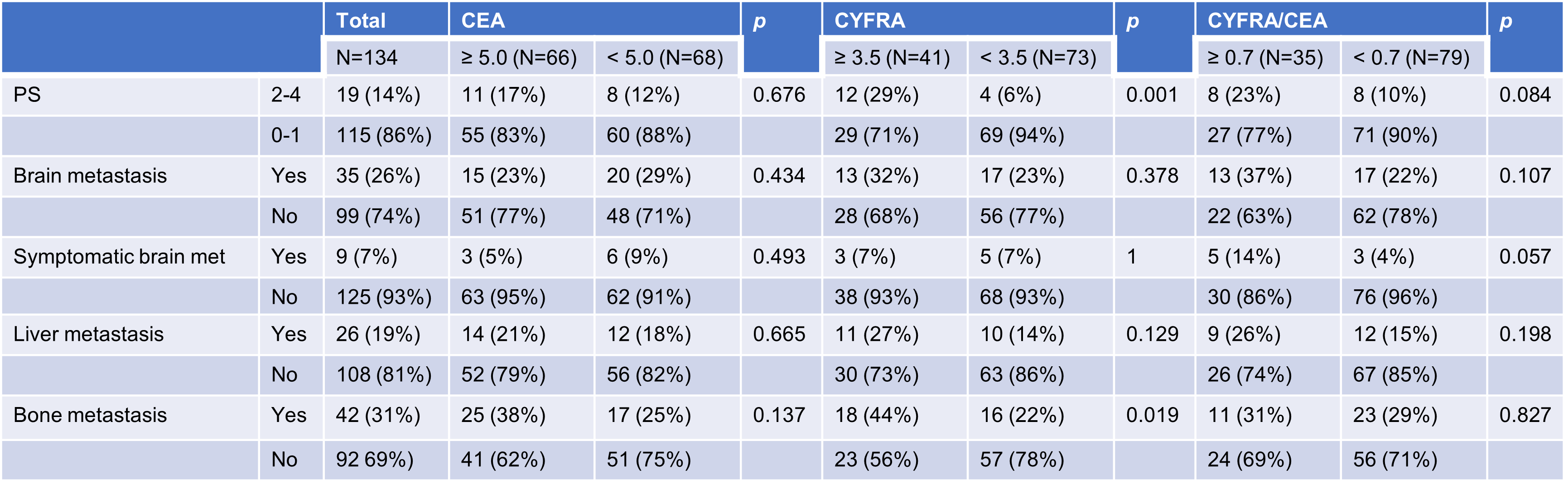


2b)


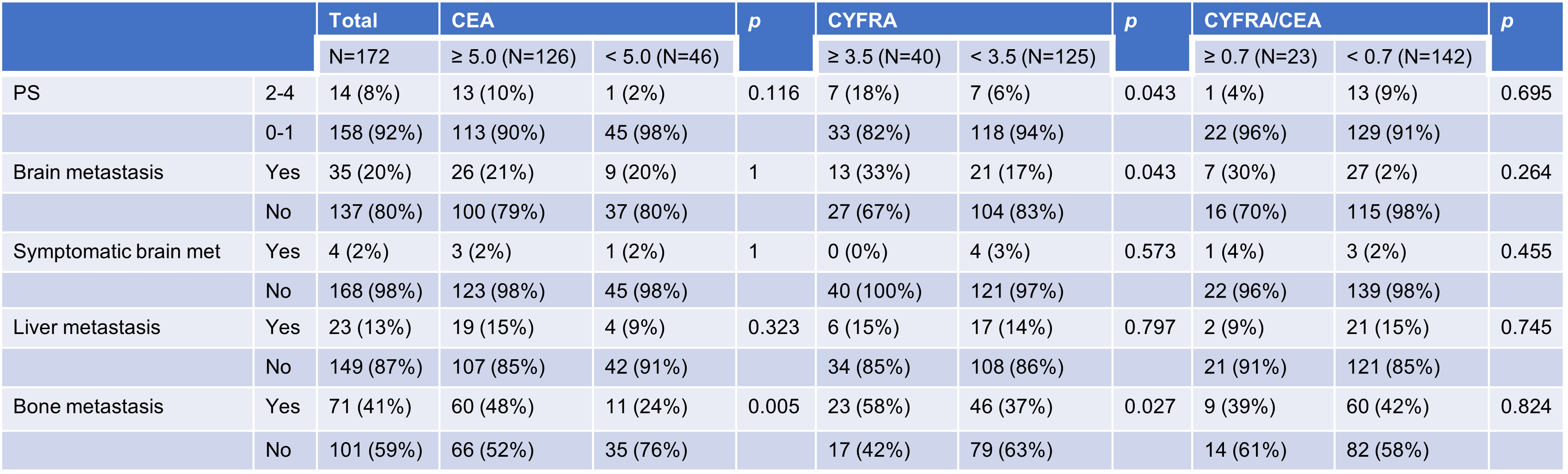


Supplementary Figure 1

The TTF following initial tyrosine kinase inhibitor (TKI) treatment was evaluated using the Kaplan–Meier method. Patients were divided into CYFRA21-1-positive or -negative groups.

(A) Kaplan–Meier curves for TTF in the ALK group without distinguishing the histological type, and (B) Kaplan–Meier curves for TTF in the EGFR group without distinguishing the histological type. (C) Kaplan–Meier curves for TTF in the ALK group in Adenocarcinoma, and (D) Kaplan–Meier curves for TTF in the EGFR group in Adenocarcinoma.

The median TTF was 260 days in cases exhibiting CYFRA21-1 positive and 979 days in cases with CYFRA21-1 negative (*p* = 0.002). In the EGFR group, the median TTF was 451 days in cases with CYFRA21-1 positive and 602 days in cases with CYFRA21-1 negative (*p* = 0.276).

The same analysis was done for the adenocarcinoma cases. In the ALK group, the median TTF with CYFRA21-1 positive was 243 days, and 979 days (*p* < 0.001) for CYFRA21-1 negative. In the EGFR group, the median TTF with CYFRA21-1 positive was 471 days and 471 days (*p* =0.768) for CYFRA21-1 negative.


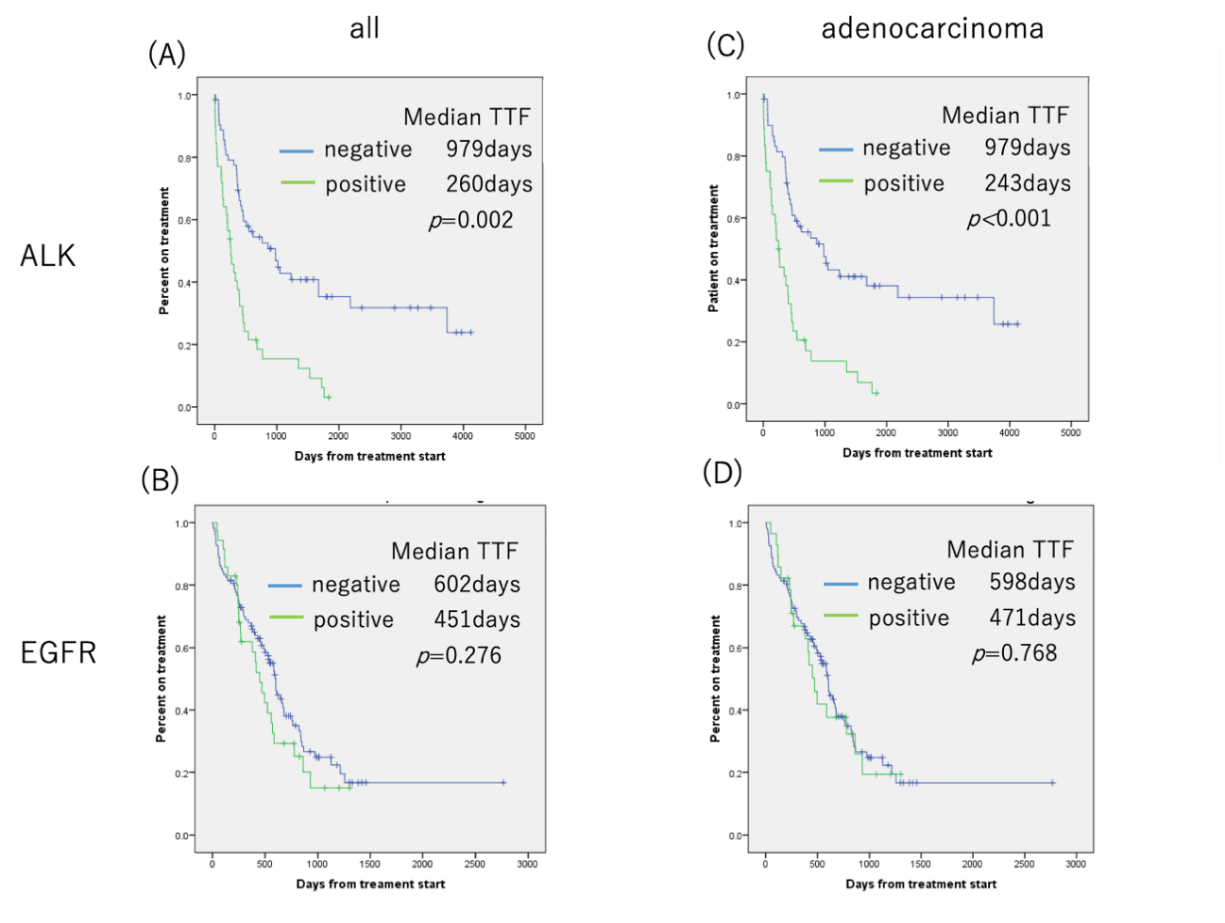


Supplementary Figure 2

The TTF following initial tyrosine kinase inhibitor (TKI) treatment was evaluated using the Kaplan–Meier method. Patients were divided into CEA-positive or -negative groups.

(A) Kaplan–Meier curves for TTF in the ALK group without distinguishing the histological type, and (B) Kaplan–Meier curves for TTF in the EGFR group without distinguishing the histological type. (C) Kaplan–Meier curves for TTF in the ALK group in Adenocarcinoma, and (D) Kaplan–Meier curves for TTF in the EGFR group in Adenocarcinoma.

The median TTF was 399 days in cases exhibiting CEA positive and 617 days in cases with CEA negative (*p* = 0.412). In the EGFR group, the median TTF was 571 days in cases with CEA positive and 544 days in cases with CEA negative (*p* = 0.595).

The same analysis was done for the adenocarcinoma cases. In the ALK group, the median TTF with CEA positive was 399 days, and 767 days (*p* = 0.230) for CEA negative. In the EGFR group, the median TTF with CEA positive was 586 days and 544 days (*p* =0.772) for CEA negative.


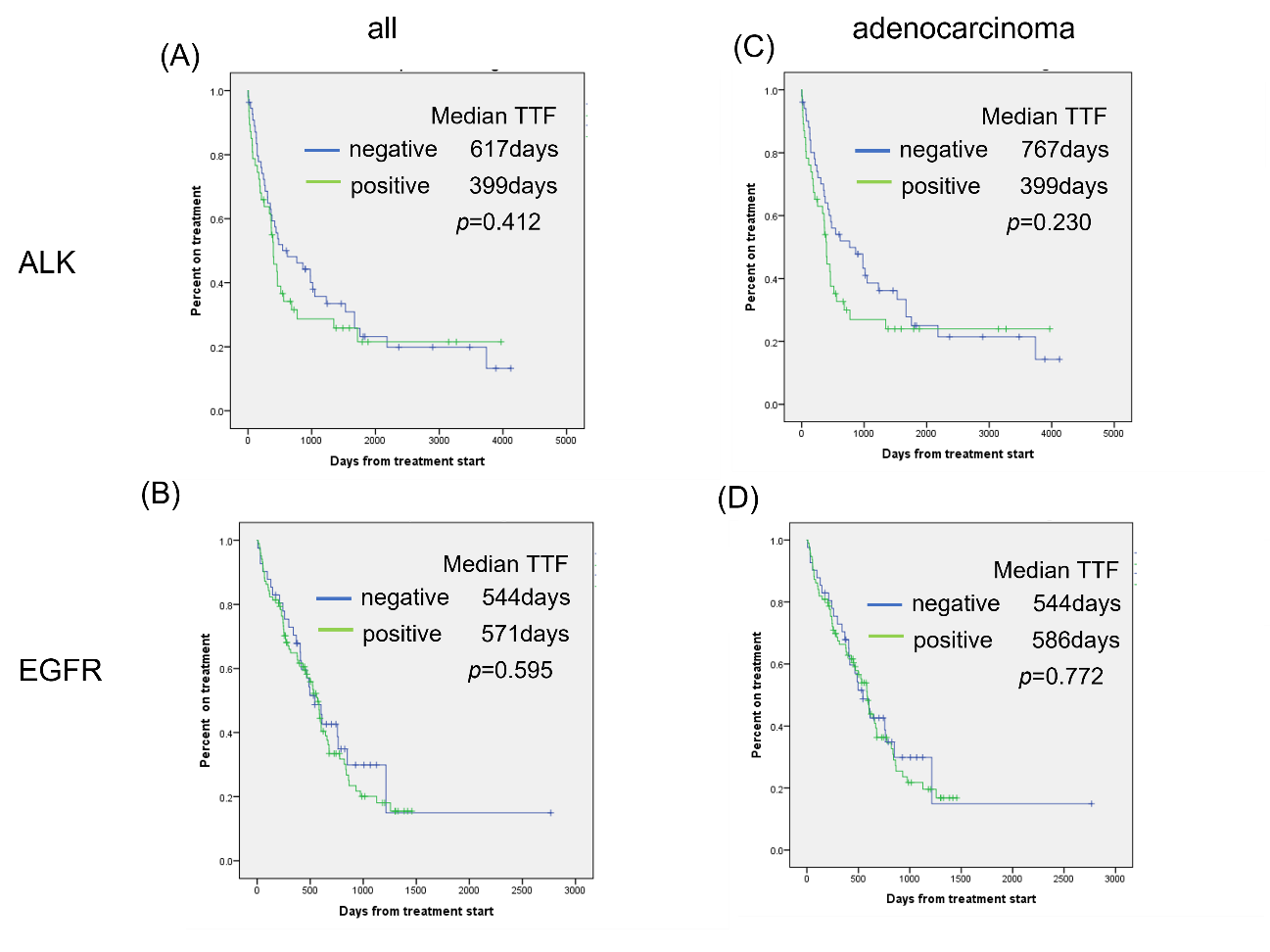

Supplement: Supplementary file 1 — DATA S1: Supplementary Information. [file TCA-15-788-s001.docx]
